# Oncogene OSTM1 Promotes Gastric-Cancer Metastasis by Modulating the Metastatic Microenvironment Through Altered Tumor-Cell Autocrine Signaling

**DOI:** 10.3390/cimb47010055

**Published:** 2025-01-16

**Authors:** Yucheng Tang, Yi Guo, Jiangyi Feng, Ziwei Wang

**Affiliations:** 1 Department of General Surgery, The First Affiliated Hospital of Chongqing Medical University, Chongqing 400016, China; 15213489737@163.com; 2 Chongqing University Central Hospital, Chongqing Emergency Medical Center, Chongqing 400014, China; fengjiangyi@126.com

**Keywords:** gastric cancer, OSTM1, S100A4, proliferation and metastasis, tumor microenvironment

## Abstract

Gastric cancer remains a malignancy with high incidence, mortality rates, and poor prognosis globally. Osteoclastogenesis-associated transmembrane protein 1 (OSTM1), a transmembrane protein overexpressed in various tumors, has unclear functions in gastric-cancer progression. This study explores OSTM1’s role in gastric-cancer proliferation and metastasis. OSTM1 expression was analyzed in gastric-cancer and adjacent tissues using immunohistochemistry and RT-qPCR. OSTM1 overexpression and knockdown cell lines were established to assess its effects on cancer-cell behavior through in vitro and in vivo experiments. Western blot and RT-qPCR were used to examine OSTM1’s regulation of S100A4 expression. OSTM1 was significantly overexpressed in gastric-cancer tissues, negatively correlating with TNM staging and overall survival. OSTM1 overexpression enhanced cancer-cell proliferation, colony formation, migration, and invasion, while its knockdown showed opposite effects. In vivo studies confirmed increased lung metastatic capability in high OSTM1-expressing cells. Mechanistically, OSTM1 positively regulated S100A4 expression, with S100A4 knockdown reducing OSTM1-enhanced metastasis. Gastric-cancer lung metastases showed higher microvascular density and α-SMA-positive fibroblast infiltration in the OSTM1 high-expression group. OSTM1 promotes gastric-cancer progression by upregulating S100A4 and modifying the tumor microenvironment through enhanced angiogenesis and fibroblast activation. OSTM1 represents a potential diagnostic and prognostic biomarker, with the OSTM1–S100A4 axis offering new therapeutic possibilities for gastric-cancer treatment.

## 1. Introduction

Gastric cancer, as one of the most common malignancies worldwide, continues to exhibit high incidence and mortality rates [1,2,3]. The development and metastasis of gastric cancer involve a complex multi-step process that includes the regulation of various genes and signaling pathways [4,5,6]. In recent years, researchers have identified several molecular markers associated with the proliferation and metastasis of gastric cancer, such as HER2 and EGFR [7,8,9]. However, early diagnosis and effective treatment of gastric cancer still pose significant challenges, especially since treatments often yield poor outcomes after metastasis [10,11]. This underscores the need for further exploration of the molecular mechanisms underlying gastric-cancer progression to identify new therapeutic targets [12,13].

OSTM1, initially discovered in the process of bone resorption, is an important transmembrane protein [14,15]. Recent studies have shown that OSTM1 is abnormally expressed in various tumors and may be associated with tumor aggressiveness and metastatic potential [15,16]. For instance, OSTM1 is highly expressed in cells and closely related to tumor aggressiveness and prognosis [17]. Additionally, S100A4, a known tumor metastasis-promoting factor, is highly expressed in various tumors and is associated with enhanced tumor aggressiveness and metastatic capability [18,19]. S100A4 facilitates tumor-cell migration and invasion by regulating interactions between the cytoskeleton and extracellular matrix [20,21].

Although existing research has revealed the potential roles of OSTM1 and S100A4 in tumor development, their specific interactions and mechanisms in gastric cancer remain unclear. Particularly, how OSTM1 influences gastric-cancer cell proliferation and metastasis through the regulation of the S100A4 signaling pathway has rarely been reported in the literature. Moreover, potential therapeutic strategies targeting this signaling pathway have not been thoroughly explored.

This study aims to investigate how OSTM1 promotes gastric-cancer proliferation and metastasis by enhancing the S100A4 signaling pathway. We analyze the expression and correlation of OSTM1 and S100A4 in gastric-cancer tissues through clinical samples and explore the regulatory effects of OSTM1 on S100A4 expression using in vitro cell experiments and in vivo animal models. Additionally, this study examines potential therapeutic strategies targeting the OSTM1/S100A4 signaling pathway, providing new insights and approaches for the treatment of gastric cancer. By investigating the roles of OSTM1 and S100A4 in gastric cancer, this study not only aids in understanding the molecular pathophysiology of gastric cancer but may also offer new molecular targets for targeted therapy, holding significant scientific value and clinical implications.

## 2. Methods

### 2.1. Specimen Collection

This study included 21 patients who underwent radical gastrectomy for gastric cancer at our hospital between February 2021 and February 2023. None of the enrolled patients had received any other treatments and had no malignant tumors originating from other tissues. Relevant clinical data from the patients were collected through the electronic medical record system of the medical records department. Paraffin blocks of gastric-cancer and adjacent tissues from gastric-cancer patients were obtained from the pathology department for immunohistochemical staining of OSTM1. This study was approved by the ethics committee of The First Affiliated Hospital of Chongqing Medical University (approval number: CQMU-2021-30), and written informed consent was obtained from all participants in accordance with the Declaration of Helsinki.

### 2.2. Immunohistochemistry

Paraffin blocks of gastric-cancer and adjacent tissues were sectioned into 4 μm slices, deparaffinized in xylene and gradient alcohol, washed with water, and boiled in sodium citrate antigen-retrieval solution for 30 min. The sections were incubated with endogenous peroxidase blocker for 30 min and a primary antibody OSTM1 (ab121327, Abcam, Waltham, MA, USA) overnight at 4 °C. After washing with PBS three times, the sections were incubated with a secondary antibody (enzyme-labeled goat anti-rabbit IgG polymer, Zhongshan Golden Bridge, Beijing, China) for 1 h and washed with PBS again. DAB chromogen (P0202, Beyotime Biotechnology, Shanghai, China) was applied, and the cell nuclei were counterstained with hematoxylin. The sections were mounted with neutral resin, and images were captured. Finally, the relative integrated optical density (IOD) values were calculated using ImageJ software (v1.8.0.345).

### 2.3. Bioinformatics Analysis

Public data from The Cancer Genome Atlas (TCGA) were used to analyze the expression levels of OSTM1 messenger RNA (mRNA) in cancer and adjacent tissues. The Kaplan-Meier Plotter website (http://kmplot.com/analysis/, accessed on 13 April 2024) was used to analyze the correlation between OSTM1 and S100A4 expression and the prognosis of gastric-cancer patients. The TNMplot database website (https://tnmplot.com/analysis/, accessed on 13 April 2024) was used to analyze the co-expression correlation between OSTM1 and S100A4 in gastric-cancer patient tissues.

### 2.4. Cell Culture

The human gastric-cancer cell lines MGC803 and AGS were purchased from the American Type Culture Collection (ATCC). Human umbilical vein endothelial cells (HUVECs) were provided by ScienCell, Carlsbad, CA, USA. The human gastric-cancer cell lines MGC803, AGS, and HUVECs were resuspended in RPMI1640 medium supplemented with 10% fetal bovine serum (FBS). The cells were cultured at 37 °C with 5% CO_2_ until the density reached 70–80%. Fetal bovine serum and RPMI1640 medium were purchased from Gibco, Grand Island, NY, USA.

### 2.5. Cell Transfection

Gastric-cancer cell lines (MGC803 and AGS) were transfected with OSTM1 overexpression (vector: GV358), interference (vector: GV248), and control lentiviral vectors (GENECHEM, Shanghai, China). After 6 h, the medium was replaced with RPMI1640 containing 10% FBS. Stable-expression cell lines were selected using a medium containing 1 μg/mL puromycin (HY-K1057, MedChemExpress, Shanghai, China) for 3 days, and gastric-cancer cells with stable overexpression and silencing of OSTM1 were obtained for subsequent studies.

### 2.6. Real-Time RT-PCR Detection of mRNA Expression in Cells

Total RNA was extracted from each group of transfected cells, and RNA integrity was determined by agarose gel electrophoresis. The concentration and purity of RNA were measured using a UV spectrophotometer. Three μg of total RNA was used as a template, and Oligo(dt) was used as a primer. The RNA was denatured at 65 °C for 10 min, and reverse transcriptase and reverse transcriptase inhibitor were added at 55 °C for 30 min and 85 °C for 5 min to obtain cDNA by reverse transcription. An appropriate amount of cDNA was then used as a template for quantitative PCR. The amplification conditions were as follows: 95 °C pre-denaturation for 5 min, 95 °C denaturation for 30 s, 60 °C annealing for 30 s, 72 °C extension for 30 s (40 cycles), 72 °C for 7 min, and stored at 4 °C.

The primer sequences are shown in Table 1. The qPCR detection primers were synthesized by Shanghai Sangon Biotech Co., Ltd. (Sangon Biotech, Shanghai, China). The blank control was used as the control group, and the 2^−ΔΔCt^ method was used to calculate the relative expression of genes.

### 2.7. Western Blot

Stable gastric-cancer cell lines were collected, and total protein was extracted using RIPA lysis buffer. Forty μg of protein was loaded into each well for electrophoresis (upper gel voltage: 80 V, 30 min; lower gel voltage: 120 V, 60 min). The proteins were transferred to a PVDF membrane (voltage: 100 V, 2 h) using the wet transfer method. The PVDF membrane was blocked with 5% skim milk for 1 h and incubated with primary antibodies overnight at 4 °C, including OSTM1 (ab121327, 1:1000, Abcam, Waltham, MA, USA), S100A4 antibody (1:1000, ab197896), and GAPDH antibody (1:5000, ab9485). The membrane was washed with TBST and incubated with a secondary antibody [HRP-labeled goat anti-rabbit IgG (H+L), Zhongshan Golden Bridge, ZB-5301, 1:3000]. Finally, ECL luminescent liquid was added, and images were captured using a multifunctional gel imaging system. The ECL luminescent liquid was purchased from MerckMillipore, Burlington, MA, USA. The fully automated chemiluminescence imaging analyzer was from Shanghai Tanon Science & Technology Co., Ltd., Shanghai, China. ImageJ (v1.8.0.345) was used to analyze the relative expression of the target proteins.

### 2.8. Co-Culture of Gastric-Cancer Cells and MRC5 Cells

A total of 1 × 10^5^ MGC803 or AGS cells were seeded into the upper chamber of a Transwell (8.0 μm, Corning, Corning, NY, USA), and 1 × 10^5^ MRC5 cells were seeded into the lower chamber. EBM cell-culture medium containing 1% FBS was added to both the upper and lower chambers, and the cells were cultured overnight at 37 °C with 5% CO_2_. The next day, the cells in the upper chamber were transferred to the lower chamber and co-cultured with MRC5 cells to simulate the tumor growth microenvironment.

### 2.9. Transwell Assay

Cell invasion was assessed using Costar Transwell plates with 8.0 mm diameter polycarbonate fibers (8 μm pore size). The bottom of the upper chamber was pre-coated with 50 μL of Matrigel (354230, Corning, Corning, NY, USA). A fresh culture medium containing 10% FBS was added to the lower chamber. The cells were then seeded onto the upper surface of the filter. The chambers were incubated under normoxic conditions for 24 h. Non-invading cells on the upper surface of the filter were removed, and the number of invading cells was observed under an inverted microscope. Five random fields were selected in each well to calculate the number of invading cells, and the relative invasion ability was calculated based on the number of invading cells in the control group.

### 2.10. Lung Metastasis Animal Model

Sixty female nude mice (18–20 g, 7 weeks old) were purchased from Beijing Vital River Laboratory Animal Technology Co., Ltd., Beijing, China (License No. SCXK(Beijing)2021-0006). Transfected human gastric-cancer cell lines were cultured in RPMI1640 medium supplemented with 10% fetal bovine serum at 37 °C and 5% CO_2_. The culture medium was changed every 2–3 days. When the cells reached 80–90% confluence, they were digested with 0.25% trypsin and centrifuged at 1000 r/min for 3 min. The supernatant was discarded, and the cells were washed three times with serum-free RPMI1640 medium. The cells were counted under a microscope and adjusted to a concentration of 5 × 107/mL in serum-free RPMI1640 medium (2 mL in total). The prepared cell suspension was stored in an ice bath and inoculated within 30 min. With the aid of a magnifying glass, the tail vein of the nude mice was accurately selected. A 33-gauge needle was inserted approximately 2 mm into the tail vein, and 100 μL of the transfected gastric-cancer cell suspension (5 × 10^6^ cells) was slowly injected. After injection, the needle was withdrawn, and a cotton ball was gently pressed for 3 s. During the model establishment process, care should be taken to avoid puncturing the tail vein of the nude mice and leakage of cancer cells.

### 2.11. HE Staining

The lungs were adequately fixed with 4% paraformaldehyde, cut open with the tumor site at the center, and embedded in paraffin. Sections (4 µm thick) were prepared and stained with HE (C0105M, Beyotime Biotechnology, Shanghai, China). The lung metastases were observed under a microscope.

### 2.12. HUVEC Tube Formation

HUVEC cells were seeded into 96-well plates (2 × 10^4^ cells/well, pre-coated with Matrigel). The tube formation in each well was observed within 24 h using an inverted microscope (Nikon, Tokyo, Japan). Three random fields were selected to calculate the degree of tube formation under different treatments, and the relative tube formation ability was calculated with reference to the tube formation in the control group.

### 2.13. EdU Staining Assay

Cells were seeded in 96-well plates (Corning, 3599) at a density of 2 × 10^4^ cells/well and cultured overnight. The culture medium containing 10 μM EdU was added and incubated at 37 °C with 5% CO_2_ for 2 h. After removing the medium, the cells were washed three times with PBS (Gibco, 10010023). The cells were then fixed with 4% paraformaldehyde (Sigma-Aldrich, 158127) at room temperature for 15 min, followed by three 5-min washes with PBS containing 0.3% Triton X-100 (Sigma-Aldrich, St. Louis, MO, USA, T8787). The Click-iT reaction mixture (containing Alexa Fluor 561 azide) was added and incubated at room temperature for 30 min in the dark. After three PBS washes, the cells were incubated with Hoechst 33342 (Thermo Fisher Scientific, Waltham, MA, USA, 62249, 1:2000 dilution) in PBS for 10 min at room temperature in the dark to stain nuclei. Finally, the cells were washed three times with PBS. Images were captured using a fluorescence microscope (Nikon, Tokyo, Japan), and the percentage of EdU-positive cells was analyzed using ImageJ software (v1.8.0.345). All EdU staining reagents were from the Click-iT EdU Alexa Fluor 561 Imaging Kit (Invitrogen, Waltham, MA, USA, C10638).

### 2.14. TUNEL Assay

Apoptosis was assessed using the Terminal deoxynucleotidyl transferase dUTP Nick End Labeling (TUNEL) assay. Cells were seeded in 24-well plates (Corning, 3526) at a density of 5 × 10^4^ cells/well and cultured overnight. After treatment, the cells were fixed with 4% paraformaldehyde (Sigma-Aldrich, 158127) for 15 min at room temperature, then permeabilized with 0.2% Triton X-100 (Sigma-Aldrich, T8787) in PBS for 5 min. TUNEL staining was performed using the In Situ Cell Death Detection Kit, Fluorescein (Roche, Basel, Switzerland, 11684795910), according to the manufacturer’s instructions. Briefly, 50 μL of TUNEL reaction mixture was added to each sample and incubated for 60 min at 37 °C in a humidified atmosphere in the dark. The cells were then washed three times with PBS. The nuclei were counterstained with DAPI (Thermo Fisher Scientific, D1306, 1:1000 dilution) for 5 min. Images were captured using a fluorescence microscope (Nikon, Tokyo, Japan) with excitation at 488 nm for TUNEL (green) and 358 nm for DAPI (blue). The percentage of TUNEL-positive cells was quantified using ImageJ software (v1.8.0.345) by analyzing at least 500 cells per condition across three independent experiments.

### 2.15. Immunofluorescence Staining

Cells were seeded on glass coverslips in 24-well plates (Corning, 3526) at a density of 4 × 10^4^ cells/well and cultured overnight. After treatment, the cells were fixed with 4% paraformaldehyde (Sigma-Aldrich, 158127) for 15 min at room temperature and then permeabilized with 0.2% Triton X-100 (Sigma-Aldrich, T8787) in PBS for 10 min. Non-specific binding was blocked with 5% bovine serum albumin (BSA, Sigma-Aldrich, A2153) in PBS for 1 h at room temperature. The cells were then incubated overnight at 4 °C with primary antibodies: rabbit anti-OSTM1 (1:200, Abcam, Waltham, MA, USA, ab199013) and mouse anti-S100A4 (1:100, Santa Cruz Biotechnology, Dallas, TX, USA, sc-53948). After washing three times with PBS, the cells were incubated with secondary antibodies: Alexa Fluor 594-conjugated goat anti-rabbit IgG (1:500, Thermo Fisher Scientific, A-11037) for OSTM1 and Alexa Fluor 488-conjugated goat anti-mouse IgG (1:500, Thermo Fisher Scientific, A-11029) for S100A4, for 1 h at room temperature in the dark. The nuclei were counterstained with DAPI (Thermo Fisher Scientific, D1306, 1:1000 dilution) for 5 min. Coverslips were mounted on glass slides using ProLong Gold Antifade Mountant (Thermo Fisher Scientific, P36934). Images were captured using a confocal laser scanning microscope (Leica TCS SP8, Wetzlar, Germany) with excitation/emission wavelengths of 594/618 nm for OSTM1 (red), 488/525 nm for S100A4 (green), and 358/461 nm for DAPI (blue). Co-localization and fluorescence intensity were analyzed using ImageJ software (v1.8.0.345).

### 2.16. Statistical Analysis

SPSS 25.0 software was used for data analysis. Measurement data were expressed as mean ± standard deviation. The *t*-test was used to compare means between two groups. One-way ANOVA was used to compare means among three groups. The chi-square test was used for comparing rates. Spearman’s method was used to calculate correlation coefficients. Kaplan–Meier curves were used to represent postoperative survival rates, and the Log-rank χ^2^ test was used to compare survival rates between groups. A value of *p* < 0.05 was considered statistically significant.

## 3. Results

### 3.1. OSTM1 Is Highly Expressed in Gastric Cancer and Is Associated with the Prognosis of Gastric-Cancer Patients

**Figure 1:**
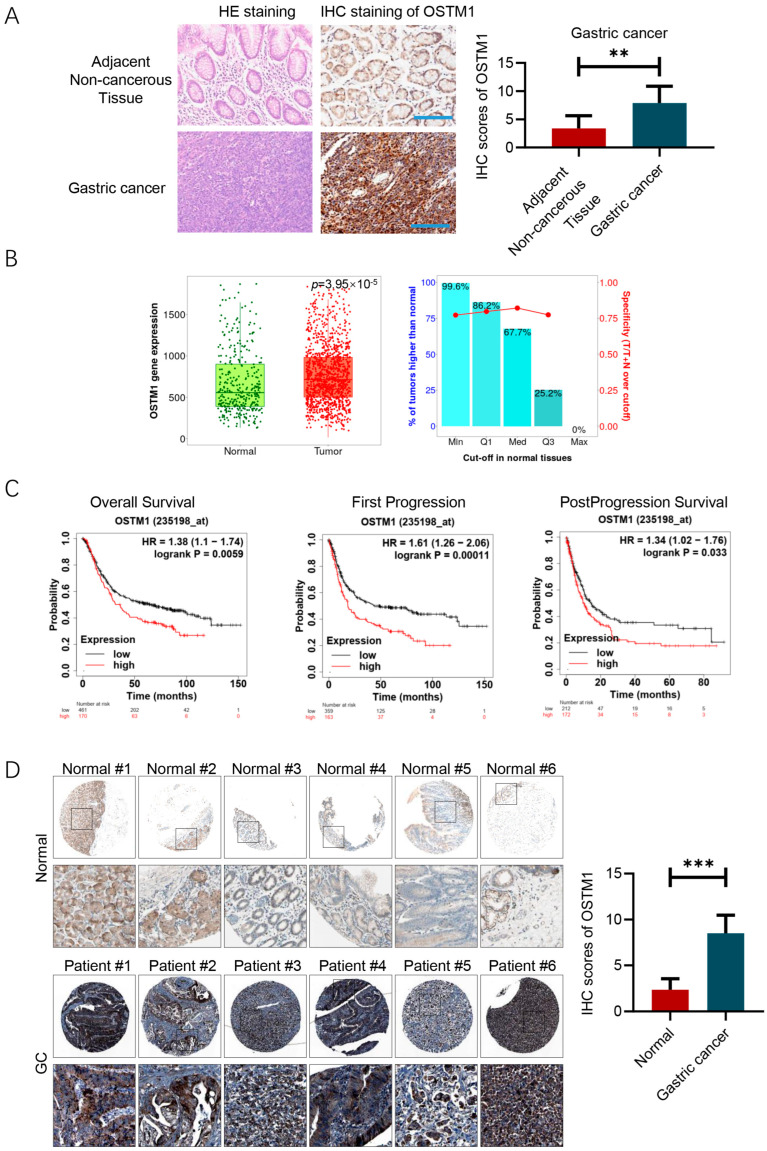
OSTM1 is highly expressed in -and associated with poor prognosis in gastric-cancer patients. (**A**) High expression of OSTM1 in gastric-cancer tissues. Scale bar: 200 μM. (**B**) OSTM1 mRNA expression data in gastric-cancer patients obtained from the TCGA database. (**C**) Survival analysis of gastric-cancer patients based on OSTM1 mRNA expression levels from the KM Plotter database. (**D**) IHC data of OSTM1 in normal or gastric-cancer tissues. The data is sourced from the Human Protein Atlas database (https://www.proteinatlas.org/). ** *p* < 0.01, *** *p* < 0.001.

In this study, we investigated the role of OSTM1 in gastric cancer and its potential mechanisms. By detecting the expression of OSTM1 in gastric-cancer tissues and adjacent tissues using immunohistochemical staining, we found that the expression of OSTM1 in gastric-cancer tissues was significantly higher than that in the adjacent normal tissues (Figure 1A). The IHC staining revealed that OSTM1 was predominantly localized in the cytoplasm of gastric-cancer cells. This suggests that OSTM1 may play a promoting role in the occurrence and development of gastric cancer. Using the TCGA database to analyze the OSTM1 mRNA expression levels in gastric-cancer patients, the results showed that the mRNA expression of OSTM1 in gastric-cancer tissues was significantly higher than that in adjacent tissues, consistent with the immunohistochemical results, further confirming the high expression trend of OSTM1 in gastric cancer (Figure 1B). By analyzing the survival data of gastric-cancer patients through the KM Plotter database, it was found that gastric-cancer patients with high OSTM1 expression had shorter overall survival, suggesting that the expression level of OSTM1 is closely related to the prognosis of gastric-cancer patients and may be a potential prognostic marker (Figure 1C). By analyzing the immunohistochemistry staining results of OSTM1 in the Human Protein Atlas database, it can also be found that OSTM1 is highly expressed in gastric-cancer tumor tissues (Figure 1D). The results of Figure 1 confirm the significantly high expression of OSTM1 in gastric cancer at both tissue and cellular levels, and the high expression of OSTM1 indicates a poor prognosis for gastric-cancer patients, suggesting that OSTM1 may be an important promoting factor in the occurrence and development of gastric cancer and has the potential to become a molecular marker for determining the prognosis of gastric cancer.

### 3.2. OSTM1 Promotes the Proliferation, Invasion, and Metastasis of Gastric-Cancer Cells

**Figure 2:**
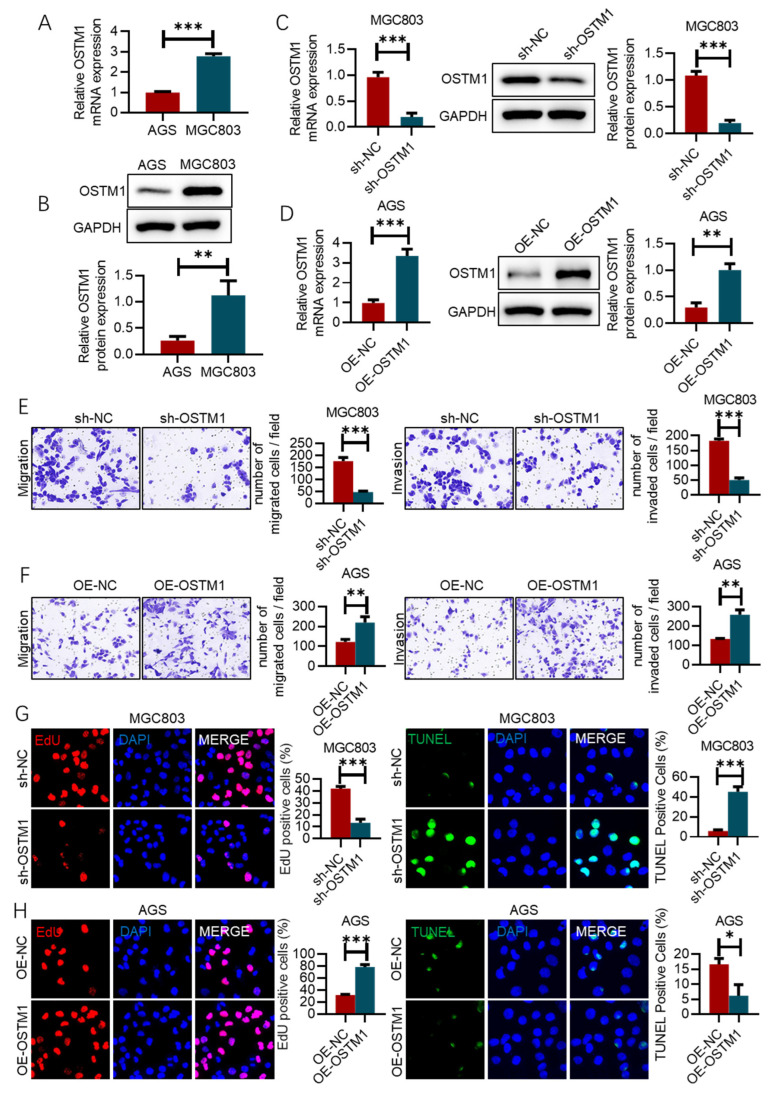
OSTM1 promotes the proliferation, invasion, and metastasis of gastric-cancer cells. (**A**) Relative mRNA expression levels of OSTM1 in gastric-cancer cell lines MGC803 and AGS, as determined by RT-qPCR. (**B**) Protein expression levels of OSTM1 in gastric-cancer cell lines MGC803 and AGS, as determined by Western blot. (**C**) Validation of OSTM1 knockdown in the human gastric-cancer cell line MGC803 by RT-qPCR and Western blot. (**D**) Validation of OSTM1 overexpression in the human gastric-cancer cell line AGS by RT-qPCR and Western blot. (**E**) Transwell migration and Matrigel invasion of MGC803 cells with OSTM1 knockdown. (**F**) Transwell migration and Matrigel invasion of AGS cells with OSTM1 overexpression. (**G**) EdU assay to assess cell proliferation capacity. (**H**) TUNEL assay to detect changes in cell apoptosis. *n* = 3, * *p* < 0.05, ** *p* < 0.01, *** *p* < 0.001.

Real-time quantitative PCR (RT-qPCR) and Western blot were used to detect the mRNA and protein expression levels of OSTM1 in two gastric-cancer cell lines, MGC803 and AGS. It was found that the expression of OSTM1 in MGC803 cells was higher than that in AGS cells (Figure 2A,B and Figure S1).

We used RNA-interference technology to knock down the expression of OSTM1 in MGC803 cells through lentivirus-mediated short hairpin RNA (shRNA). The results of RT-qPCR and Western blot showed that compared with the control group (sh-NC), the mRNA and protein expression levels of OSTM1 in the OSTM1 knockdown group (sh-OSTM1) were significantly reduced, indicating successful OSTM1 knockdown (Figure 2C). Subsequently, an OSTM1 overexpression plasmid was constructed and transfected into AGS cells. The results of RT-qPCR and Western blot showed that compared with the control group (OE-NC), the mRNA and protein expression levels of OSTM1 in the OSTM1 overexpression group (OE-OSTM1) were significantly increased, confirming the successful construction of OSTM1 overexpression (Figure 2D). The effects of OSTM1 on the migration and invasion abilities of MGC803 cells were evaluated through Transwell chamber and Matrigel invasion assays. The results showed that after OSTM1 knockdown, the number of MGC803 cells passing through the Transwell chamber membrane was significantly reduced, and the number of invading cells passing through the Matrigel matrix was also significantly decreased, indicating that the knockdown of OSTM1 can inhibit the migration and invasion abilities of MGC803 cells (Figure 2E). Conversely, after overexpression of OSTM1, the number of AGS cells passing through the Transwell chamber membrane and the number of invading cells passing through the Matrigel matrix were significantly increased, indicating that the overexpression of OSTM1 can promote the migration and invasion abilities of AGS cells (Figure 2F). EdU assay results show that the overexpression of OSTM1 promotes the proliferation of AGS cells, while silencing OSTM1 inhibits the proliferation capacity of MGC803 cells (Figure 2G). TUNEL assay results indicate that silencing OSTM1 induces apoptosis in MGC803 cells, whereas the overexpression of OSTM1 reduces the occurrence of apoptosis in AGS cells (Figure 2H).

### 3.3. OSTM1 Promotes Metastasis and Angiogenesis in Gastric Cancer

**Figure 3:**
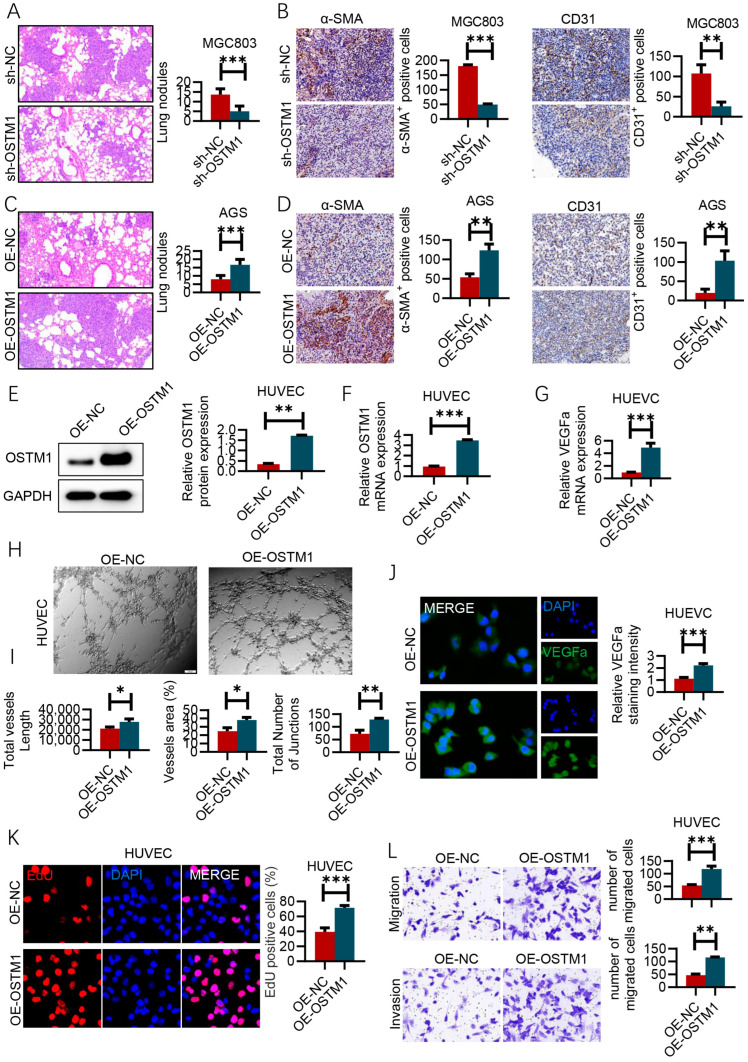
OSTM1 promotes metastasis and angiogenesis in gastric cancer. (**A**) Quantification and representative images of lung metastatic nodules after tail-vein injection of MGC803 cells with OSTM1 knockdown (*n* = 6 mice per group). (**B**) Immunohistochemistry (IHC) analysis of α-SMA^+^ and CD31^+^ cells in lung metastases of animal models after tail-vein injection of MGC803 cells with OSTM1 knockdown. (**C**) Quantification and representative images of lung metastatic nodules after tail-vein injection of AGS cells with OSTM1 overexpression (*n* = 6 mice per group). (**D**) IHC analysis of α-SMA^+^ and CD31^+^ cells in lung metastases of animal models after tail-vein injection of AGS cells with OSTM1 overexpression. (**E**,**F**) Western blot (**E**) and RT-PCR (**F**) analysis confirming OSTM1 overexpression in HUVECs. (**G**) Relative mRNA expression levels of VEGFA in HUVECs under the respective treatment conditions. (**H**) Tube formation of HUVECs with OSTM1 overexpression. (**I**) Assessment of tube-formation ability of HUVECs by tube length, percentage of tube area, and number of vascular connection points. (**J**) IF staining of VEGFA in HUVECs under the respective treatment conditions. (**K**) EdU assay to assess cell proliferation capacity. (**L**) Transwell assay to evaluate cell migration and invasion abilities. *n* = 3, * *p* < 0.05, ** *p* < 0.01, *** *p* < 0.001.

To study the effect of OSTM1 on the in vivo tumorigenic ability of gastric-cancer cells, we injected MGC803 cells with OSTM1 knockdown into nude mice via the tail vein and observed lung metastasis. The results showed that compared with the control group, the number of metastatic foci in the lungs of mice in the OSTM1 knockdown group was significantly reduced, suggesting that the knockdown of OSTM1 can inhibit the lung-metastasis ability of MGC803 cells in vivo (Figure 3A). α-SMA and CD31 are markers of vascular smooth-muscle cells and endothelial cells, respectively. By detecting the expression levels of α-SMA^+^ and CD31^+^ cells in lung metastases using immunohistochemistry, the vascular density in tumor tissues can be assessed, reflecting the angiogenic ability of tumors. The results showed that compared with the control group (sh-NC), the number of α-SMA^+^ and CD31^+^ cells in lung metastases of OSTM1 knockdown-group (sh-OSTM1) mice was significantly reduced, suggesting that the knockdown of OSTM1 could inhibit angiogenesis in gastric-cancer lung metastases (Figure 3B). Conversely, after injecting AGS cells overexpressing OSTM1 into nude mice via the tail vein, the number of metastatic foci in the lungs of mice in the OSTM1 overexpression group was significantly higher than that in the control group, indicating that the overexpression of OSTM1 can promote the lung-metastasis ability of AGS cells in vivo (Figure 3C). Compared with the control group (OE-NC), the number of α-SMA^+^ and CD31^+^ cells in lung metastases of OSTM1 overexpression-group (OE-OSTM1) mice was significantly increased, indicating that the overexpression of OSTM1 could promote angiogenesis in gastric-cancer lung metastases (Figure 3D). After confirming OSTM1 overexpression in HUVECs by Western blot and RT-PCR (Figure 3E,F), we found increased VEGFA mRNA expression levels (Figure 3G). The in vitro tube-formation assay is an important method to evaluate the angiogenic ability of endothelial cells. The results showed that after the overexpression of OSTM1, the number of tube structures formed by HUVECs was significantly higher than that of the control group, indicating that OSTM1 overexpression could promote the angiogenic ability of HUVECs (Figure 3H). Tube length, tube area percentage, and the number of vascular connection points are important indicators for quantitative evaluation of the in vitro tube-formation ability of endothelial cells. The results showed that compared with the control group, the tube length, tube area percentage, and the number of vascular connection points formed by HUVECs in the OSTM1 overexpression group were significantly increased, further confirming that OSTM1 overexpression could promote the angiogenic ability of HUVECs (Figure 3I). IF staining results showed that the expression level of VEGFA in HUVECs of the OSTM1 overexpression group was significantly higher than that of the control group, suggesting that OSTM1 might promote angiogenesis of endothelial cells by upregulating the expression of VEGFA (Figure 3J). Furthermore, the overexpression of OSTM1 can promote the proliferation, invasion, and migration abilities of HUVEC cells (Figure 3K,L).

### 3.4. OSTM1 Promotes Fibroblast Activation in Gastric-Cancer Lung Metastasis

**Figure 4:**
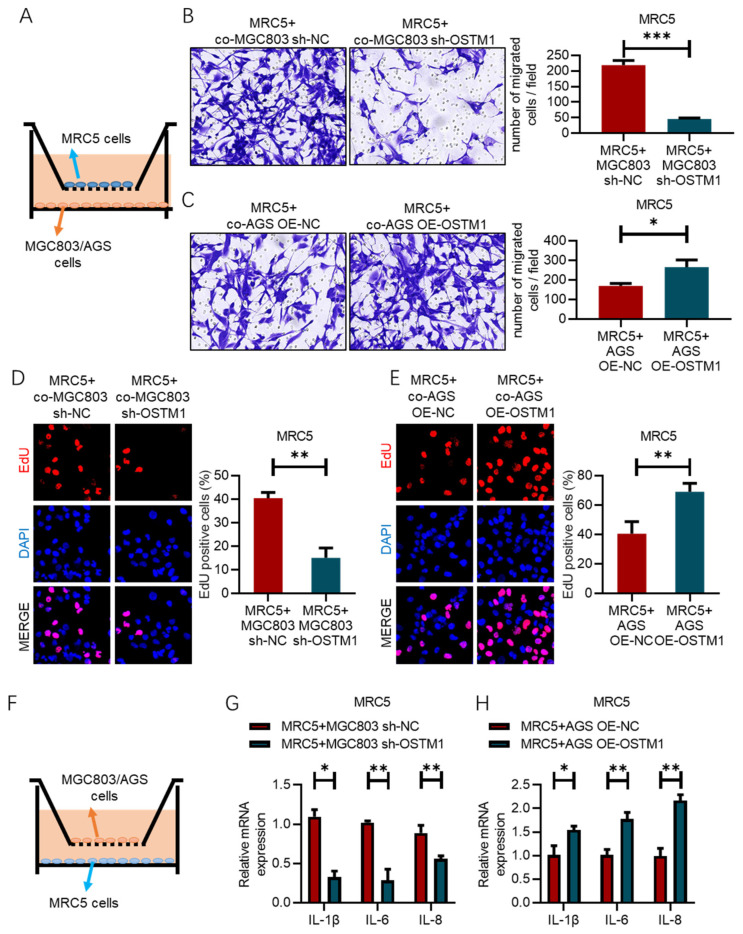
OSTM1 promotes fibroblast activation in gastric-cancer lung metastasis. (**A**) Schematic diagram of the Transwell migration assay. (**B**) Enhanced migration ability of MRC5 cells co-cultured with OSTM1-overexpressing AGS cells. (**C**) Reduced migration ability of MRC5 cells co-cultured with OSTM1-downregulated MGC803 cells. (**D**,**E**) EdU assay to assess cell proliferation capacity of MRC5 cells. (**F**) Schematic diagram of the Transwell co-culture assay. (**G**) Relative mRNA expression of inflammatory factors IL-1β, IL6, and IL8 in MRC5 cells co-cultured with MGC803 cells, as determined by RT-qPCR. (**H**) Relative mRNA expression of inflammatory factors IL-1β, IL6, and IL8 in MRC5 cells co-cultured with AGS cells, as determined by RT-qPCR. *n* = 3, * *p* < 0.05, ** *p* < 0.01, *** *p* < 0.001.

In the tumor microenvironment, fibroblasts are the most abundant cell type besides tumor cells. Tumor cells can recruit and activate fibroblasts by secreting various cytokines and chemokines, transforming them into cancer-associated fibroblasts (CAFs). In turn, activated CAFs can promote tumor-cell proliferation, invasion, and metastasis by secreting extracellular matrix, cytokines, and chemokines. Therefore, investigating the impact of OSTM1 on fibroblast activation during gastric-cancer lung metastasis is of great significance. Figure 4A illustrates the schematic diagram of the Transwell migration assay. MRC5 lung fibroblasts were seeded in the upper chamber of the Transwell insert, while gastric-cancer cells were seeded in the lower chamber. The effect of gastric-cancer cells on MRC5 migration ability was assessed by quantifying the number of MRC5 cells that migrated to the lower surface of the insert. The results in Figure 4B show that, compared to the control group (AGS OE-NC+MRC5), the OSTM1 overexpression group (AGS OE-OSTM1+MRC5) significantly enhanced the migration ability of MRC5 cells. This suggests that OSTM1-overexpressing gastric-cancer cells can promote the migration of MRC5 lung fibroblasts. Conversely, the results in Figure 4C demonstrate that compared to the control group (MGC803 sh-NC+MRC5), the OSTM1 knockdown group (MGC803 sh-OSTM1+MRC5) markedly reduced the migration ability of MRC5 cells. This further confirms that the expression level of OSTM1 positively correlates with the ability of gastric-cancer cells to promote MRC5 migration. Figure 4D presents the schematic diagram of the Transwell co-culture experiment. Gastric-cancer cells were seeded in the upper chamber of the Transwell insert, while MRC5 cells were seeded in the lower chamber. The trend changes in the EdU assay results are consistent with the Transwell assay results (Figure 4D,E). The effect of gastric-cancer cells on MRC5 activation was evaluated by detecting the expression of inflammatory factors in MRC5 cells. The experimental results show that compared to the control group (MGC803 sh-NC+MRC5), the OSTM1 knockdown group (MGC803 sh-OSTM1+MRC5) exhibited significantly decreased mRNA expression levels of inflammatory factors such as IL-1β, IL6, and IL8 in MRC5 cells (Figure 4F,G). This indicates that OSTM1 knockdown can inhibit the inflammatory activation of MRC5 induced by gastric-cancer cells. Compared to the control group (AGS OE-NC+MRC5), the OSTM1 overexpression group (AGS OE-OSTM1+MRC5) showed markedly increased mRNA expression levels of IL-1β, IL6, and IL8 in MRC5 cells. This demonstrates that OSTM1 overexpression can promote the inflammatory activation of MRC5 induced by gastric-cancer cells (Figure 4H). The above experimental results suggest that OSTM1 can play a crucial role in the microenvironment of gastric-cancer lung metastasis by promoting the interaction between gastric-cancer cells and MRC5 lung fibroblasts, enhancing MRC5 migration and inflammatory activation.

### 3.5. OSTM1 Positively Regulates S100A4 Expression

**Figure 5:**
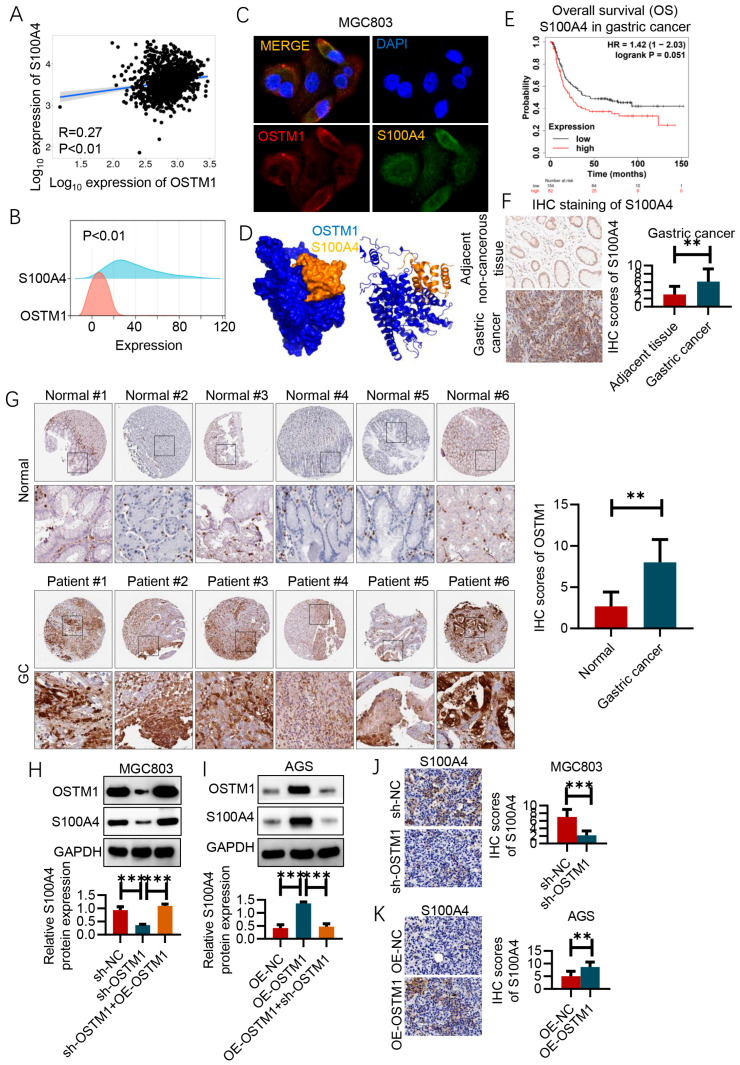
OSTM1 positively regulates S100A4 expression. (**A**) Correlation analysis of S100A4 and OSTM1 mRNA expression in metastatic lesions of gastric-cancer patients. (**B**) Ridgeline plot analysis of OSTM1 and S100A4 in the gastric-cancer data analysis results. (**C**) Immunofluorescence staining confirmed the co-localization of OSTM1 and S100A4 within cells. (**D**) Protein–protein docking results revealed the interaction conformation between OSTM1 and S100A4. (**E**) Overall survival (OS) analysis of gastric-cancer patients based on S100A4 mRNA expression levels from the KM Plotter database. (**F**) Immunohistochemical staining results of S100A4 in tumor tissues and adjacent tissues collected from patients within the group. (**G**) Immunohistochemical staining results of S100A4 in normal and gastric-cancer (GC) tissues from the THPA database. (**H**) Assessment of the effect of OSTM1 on S100A4 protein levels in MGC803 cells by Western blot. (**I**) Assessment of the effect of OSTM1 on S100A4 protein levels in AGS cells by Western blot. (**J**) Immunohistochemistry (IHC) analysis of S100A4 in lung metastases of animal models after tail-vein injection of MGC803 cells with OSTM1 knockdown. (**K**) Immunohistochemistry (IHC) analysis of S100A4 in lung metastases of animal models after tail-vein injection of AGS cells with OSTM1 overexpression. *n* = 6, ** *p* < 0.01, *** *p* < 0.001.

The results in Figure 5A indicate that in the metastatic lesions of gastric-cancer patients, the mRNA expression levels of S100A4 and OSTM1 are positively correlated (r = 0.27, *p* < 0.01).

The ridgeline plot results show that OSTM1 and S100A4 have a co-expression trend in the gastric-cancer data analysis (Figure 5B).

Immunofluorescence staining confirmed that OSTM1 and S100A4 are co-localized within cells (Figure 5C). Protein–protein docking results revealed the interaction conformation between OSTM1 and S100A4 (Figure 5D).

This suggests that OSTM1 may positively regulate S100A4 expression, and the two may play a synergistic role in the metastasis of gastric cancer. According to the analysis of the KM Plotter database, gastric-cancer patients with high S100A4 mRNA expression have shorter overall survival (OS). This indicates that high expression of S100A4 may be an indicator of poor prognosis in gastric-cancer patients, suggesting that S100A4 may promote the progression of gastric cancer (Figure 5E). Immunohistochemical staining results also show that S100A4 is highly expressed in gastric-cancer tumor tissues (Figure 5F,G).

In the in vitro experiments, the results in Figure 5H show that after knocking down OSTM1 in MGC803 cells (sh-OSTM1 group), the protein expression level of S100A4 decreases, while overexpressing OSTM1 on the basis of OSTM1 knockdown (sh-OSTM1+OE-OSTM1 group) restores the protein expression of S100A4 to the level of the control group (sh-NC group). This directly confirms that OSTM1 positively regulates the protein expression of S100A4. Correspondingly, the results in Figure 5I show that after overexpressing OSTM1 in AGS cells (OE-OSTM1 group), the protein expression level of S100A4 increases, while knocking down OSTM1 on the basis of OSTM1 overexpression (OE-OSTM1+sh-OSTM1 group) restores the protein expression of S100A4 to the level of the control group (OE-NC group). This further verifies the conclusion that OSTM1 positively regulates S100A4 protein expression. Finally, in animal experiments, the results in Figure 5J show that after tail-vein injection of MGC803 cells with OSTM1 knockdown (sh-OSTM1 group), the S100A4 protein expression (detected by immunohistochemistry) in the formed gastric-cancer lung metastases is lower than that in the control group (sh-NC group). Conversely, the results in Figure 5K show that after tail-vein injection of AGS cells with OSTM1 overexpression (OE-OSTM1 group), the S100A4 protein expression in the formed gastric-cancer lung metastases is higher than that in the control group (OE-NC group). These in vivo experimental results support the inference that OSTM1 positively regulates S100A4 expression, which may affect gastric-cancer lung metastasis. In summary, this study systematically confirms that OSTM1 may play an important role in the progression and metastasis of gastric cancer by positively regulating S100A4 expression from multiple levels, including gastric-cancer patient samples, in vitro cells, and animal models. S100A4, as a key factor promoting tumor invasion and metastasis, is regulated by OSTM1, providing new clues and potential therapeutic targets for elucidating the mechanism of gastric-cancer metastasis.

### 3.6. S100A4 Mediates the Function of OSTM1 in Gastric-Cancer Lung Metastasis

**Figure 6:**
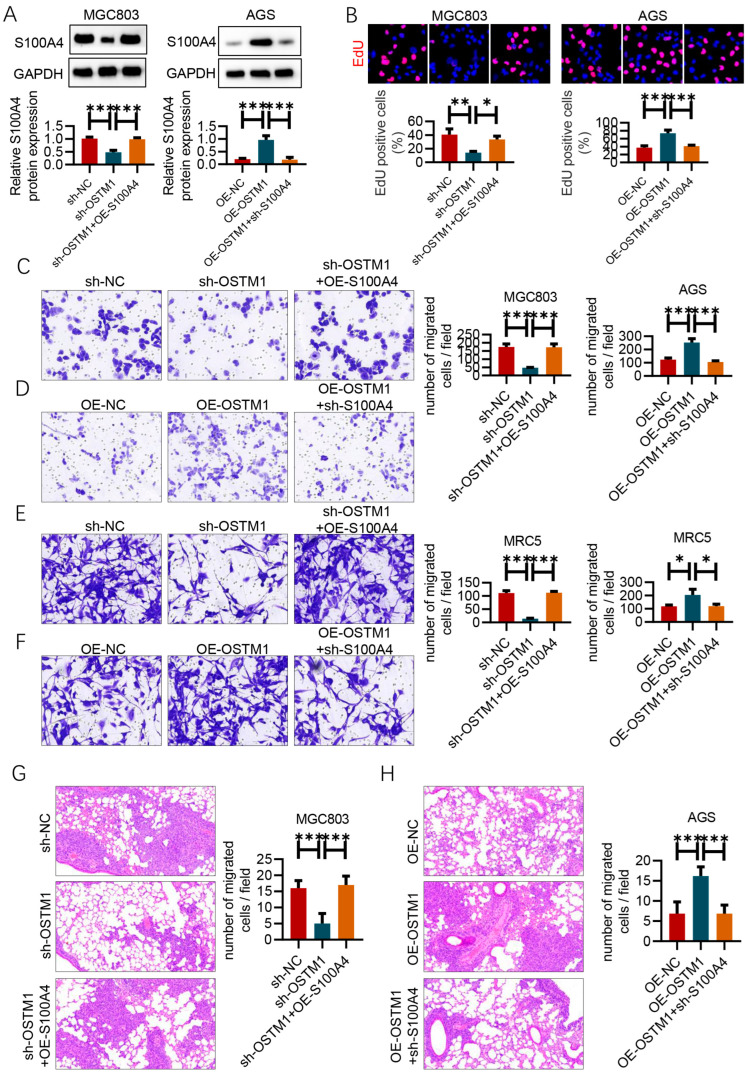
S100A4 mediates the function of OSTM1 in gastric-cancer lung metastasis. (**A**) Validation of S100A4 overexpression in the human gastric-cancer cell line MGC803 and AGS by Western blot analysis. (**B**) EdU assay to assess cell proliferation capacity. (**C**) Transwell invasion assay of MGC803 cells with different treatments (sh-NC, sh-OSTM1, sh-OSTM1+OE-S100A4). (**D**) Transwell invasion assay of AGS cells with different treatments (OE-NC, OE-OSTM1, OE-OSTM1+sh-S100A4). (**E**) Transwell migration assay of MRC5 cells co-cultured with MGC803 cells with different treatments (sh-NC, sh-OSTM1, sh-OSTM1+OE-S100A4). (**F**) Transwell migration assay of MRC5 cells co-cultured with AGS cells with different treatments (OE-NC, OE-OSTM1, OE-OSTM1+sh-S100A4). (**G**) Quantification and representative images of lung metastatic nodules after tail-vein injection of MGC803 cells with different treatments (sh-NC, sh-OSTM1, sh-OSTM1+OE-S100A4); *n* = 6 mice per group. (**H**) Quantification and representative images of lung metastatic nodules after tail-vein injection of AGS cells with different treatments (OE-NC, OE-OSTM1, OE-OSTM1+sh-S100A4); *n* = 6 mice per group. *n* = 6, * *p* < 0.05, ** *p* < 0.01, *** *p* < 0.001.

After knocking down OSTM1 and overexpressing S100A4 in MGC803 cells and overexpressing OSTM1 and knocking down S100A4 in AGS cells, Western blot results confirmed the effectiveness of the corresponding gene manipulations (Figure 6A).

EdU assay results showed that in MGC803 cells, the proliferation rate in the sh-OSTM1 group was significantly lower than that in the control sh-NC group, but after overexpressing S100A4 on the basis of sh-OSTM1, the proliferation rate recovered to the control level. In AGS cells, the proliferation rate in the OE-OSTM1 group was significantly higher than that in the control OE-NC group, but after knocking down S100A4 on the basis of OE-OSTM1, the proliferation rate decreased to the control level (Figure 6B).

**Figure 7:**
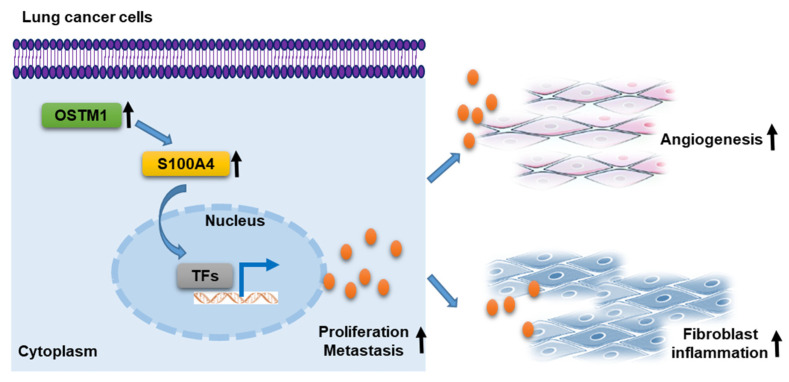
Schematic diagram depicting the roles of OSTM1 in gastric-cancer metastasis.

Transwell invasion assay results showed that in MGC803 cells, the number of invading cells in the sh-OSTM1 group was significantly lower than that in the control sh-NC group, but after overexpressing S100A4 on the basis of sh-OSTM1, the number of invading cells recovered to the control level (Figure 6C). In AGS cells, the number of invading cells in the OE-OSTM1 group was significantly higher than that in the control OE-NC group, but after knocking down S100A4 on the basis of OE-OSTM1, the number of invading cells decreased to the control level (Figure 6D). This suggests that the expression level of S100A4 affects the regulatory effect of OSTM1 on the invasive ability of gastric-cancer cells. Furthermore, the above gene-manipulated gastric-cancer cells were co-cultured with MRC5 fibroblasts. Transwell migration assay results showed that the number of migrated MRC5 cells co-cultured with MGC803–sh-OSTM1 was significantly lower than that in the MGC803–sh-NC group, but after overexpressing S100A4 on the basis of sh-OSTM1, the number of migrated MRC5 cells recovered to the control level (Figure 6E). The number of migrated MRC5 cells co-cultured with AGS–OE-OSTM1 was significantly higher than that in the AGS–OE-NC group, but after knocking down S100A4 on the basis of OE-OSTM1, the number of migrated MRC5 cells decreased to the control level (Figure 6F). This suggests that S100A4 mediates the chemotactic effect of OSTM1-overexpressing gastric-cancer cells on fibroblasts. Finally, nude mouse tail-vein injection metastasis experiments showed that mice injected with MGC803-sh-OSTM1 cells had significantly fewer lung metastatic nodules than mice injected with MGC803-sh-NC cells, but after overexpressing S100A4 on the basis of sh-OSTM1, the number of lung metastatic nodules recovered to the control level (Figure 6G). Mice injected with AGS–OE-OSTM1 cells had significantly more lung metastatic nodules than mice injected with AGS–OE-NC cells, but after knocking down S100A4 on the basis of OE-OSTM1, the number of lung metastatic nodules decreased to the control level (Figure 6H). This suggests that S100A4 mediates the role of OSTM1 in promoting gastric-cancer lung metastasis. In conclusion, S100A4 is a key molecule for OSTM1 to exert its promoting effect on gastric-cancer invasion and metastasis. It mediates the regulation of OSTM1 on the invasive ability of gastric-cancer cells and the chemotactic effect of OSTM1-overexpressing gastric-cancer cells on fibroblasts (Figure 7).

## 4. Discussion

In this study, we investigated the role of OSTM1 in gastric-cancer proliferation and metastasis through the enhancement of the S100A4 signaling pathway. The results demonstrate that high expression of OSTM1 in gastric-cancer tissues is significantly associated with the invasiveness and metastatic potential of gastric cancer, and this effect may be mediated through the regulation of the S100A4 signaling pathway.

Firstly, our study found that the expression level of OSTM1 in gastric-cancer tissue samples was significantly higher than that in normal gastric tissues, which is consistent with previous reports [17]. This suggests that OSTM1 may be a molecular marker generally involved in tumor development. Further mechanistic studies showed that OSTM1 could significantly enhance the mRNA and protein expression levels of S100A4. S100A4 is a known protein that promotes tumor invasion and metastasis by regulating the interaction between the cytoskeleton and extracellular matrix, enhancing the migration ability of tumor cells [22,23]. In gastric-cancer cell models, cells overexpressing OSTM1 exhibited higher invasive and migratory capabilities, while these effects were significantly attenuated after S100A4 knockdown. These results indicate that OSTM1 may promote gastric-cancer invasion and metastasis by regulating S100A4.

Furthermore, we also explored the role of OSTM1 and S100A4 in the gastric-cancer microenvironment. The study found that OSTM1 directly acts on tumor cells but also indirectly promotes tumor proliferation and metastasis by influencing cells in the tumor microenvironment, such as fibroblasts and endothelial cells [24,25]. For example, OSTM1 can enhance the activity of fibroblasts, promoting their secretion of factors that stimulate tumor growth and angiogenesis. This ability to “educate” surrounding cells is an important aspect of tumor microenvironment regulation and is one of the potential mechanisms by which OSTM1 promotes gastric-cancer development.

Our study reveals the oncogenic role of OSTM1 in gastric cancer, which is consistent with previous reports in other tumors. However, it is worth noting that these studies did not elucidate the downstream molecular mechanisms of OSTM1. Our study further reveals that S100A4 is a key effector molecule of OSTM1. S100A4 is an important member of the S100 calcium-binding protein family and has been shown to promote tumor progression in various tumors [26,27]. A recent study found that S100A4 can promote the invasion and metastasis of gastric-cancer cells by upregulating the expression of MMP9 [28,29,30]. Therefore, we speculate that the OSTM1–S100A4 axis may play a key role in the progression of gastric cancer.

There are several limitations to this study. Firstly, although we have preliminarily revealed the important role of the OSTM1–S100A4 axis in gastric-cancer progression, the deeper molecular mechanisms of how OSTM1 regulates S100A4 expression remain unclear, requiring further mechanistic studies for elucidation. This study is mainly based on cellular and animal models and lacks validation with large-scale clinical samples. Moreover, whether OSTM1 regulates other signaling pathways during the development and progression of gastric cancer is also worth further exploration. Future research directions mainly include the following: In-depth elucidation of the molecular mechanisms by which OSTM1 regulates S100A4 expression. More gastric-cancer tissue specimens need to be collected to assess the correlation between OSTM1 expression and patient prognosis and to explore its potential as a molecular marker. The role of OSTM1 in gastric-cancer development should be investigated using genetically engineered mouse models. Additionally, potential therapeutic strategies targeting OSTM1 and S100A4, such as small molecule inhibitors or antibody drugs, are also worth further exploration.

In summary, our study reveals the key role of OSTM1 in promoting gastric-cancer proliferation and metastasis by enhancing the S100A4 signaling pathway, providing new insights for molecular targeted therapy of gastric cancer. These findings not only enhance our understanding of the pathogenic mechanisms of gastric cancer but also provide a scientific basis for developing new therapeutic strategies.

## 5. Conclusions

Through this study, we discovered that osteoclastogenesis-associated transmembrane protein 1 (OSTM1) is highly expressed in gastric-cancer tissues, and its expression level is closely related to the clinical staging and prognosis of gastric-cancer patients. Further in vivo and in vitro experiments confirmed that OSTM1 promotes the proliferation, migration, and invasion abilities of gastric-cancer cells by enhancing the expression and activity of S100A4. In nude mouse models, gastric-cancer cells with high OSTM1 expression also exhibited stronger tumorigenic and metastatic capabilities. In summary, this study reveals the important role of the OSTM1–S100A4 axis in the development and progression of gastric cancer, providing new potential markers and intervention targets for the diagnosis and treatment of gastric cancer.

## Figures and Tables

**Table 1:** Sequences of mRNA primers.

Gene	Forward (5′-3′)	Reverse (5′-3′)
*VEGFA*	TTGCCTTGCTGCTCTACCTCCA	GATGGCAGTAGCTGCGCTGATA
*IL1B*	CCACAGACCTTCCAGGAGAATG	GTGCAGTTCAGTGATCGTACAGG
*IL6*	AGACAGCCACTCACCTCTTCAG	TTCTGCCAGTGCCTCTTTGCTG
*IL8*	GAGAGTGATTGAGAGTGGACCAC	CACAACCCTCTGCACCCAGTTT
*GAPDH*	GTCTCCTCTGACTTCAACAGCG	ACCACCCTGTTGCTGTAGCCAA

## Data Availability

Data included in the article/Supplementary Materials or referenced in the article.

## Supplementary Materials

The following supporting information can be downloaded at: https://www.mdpi.com/1467-3045/47/1/55/s1, Figure S1: Uncropped gels for Western Blots in figures.
