# Supplementary material for: Oncogene OSTM1 Promotes Gastric-Cancer Metastasis by Modulating the Metastatic Microenvironment Through Altered Tumor-Cell Autocrine Signaling
Source: Curr Issues Mol Biol. 2025 Jan 16;47(1):55. doi: 10.3390/cimb47010055 (PMC11840279; doi:10.3390/cimb47010055)
Supplement: Supplementary file 1 [file cimb-47-00055-s001.zip › cimb-3353273-supplementary.pdf]

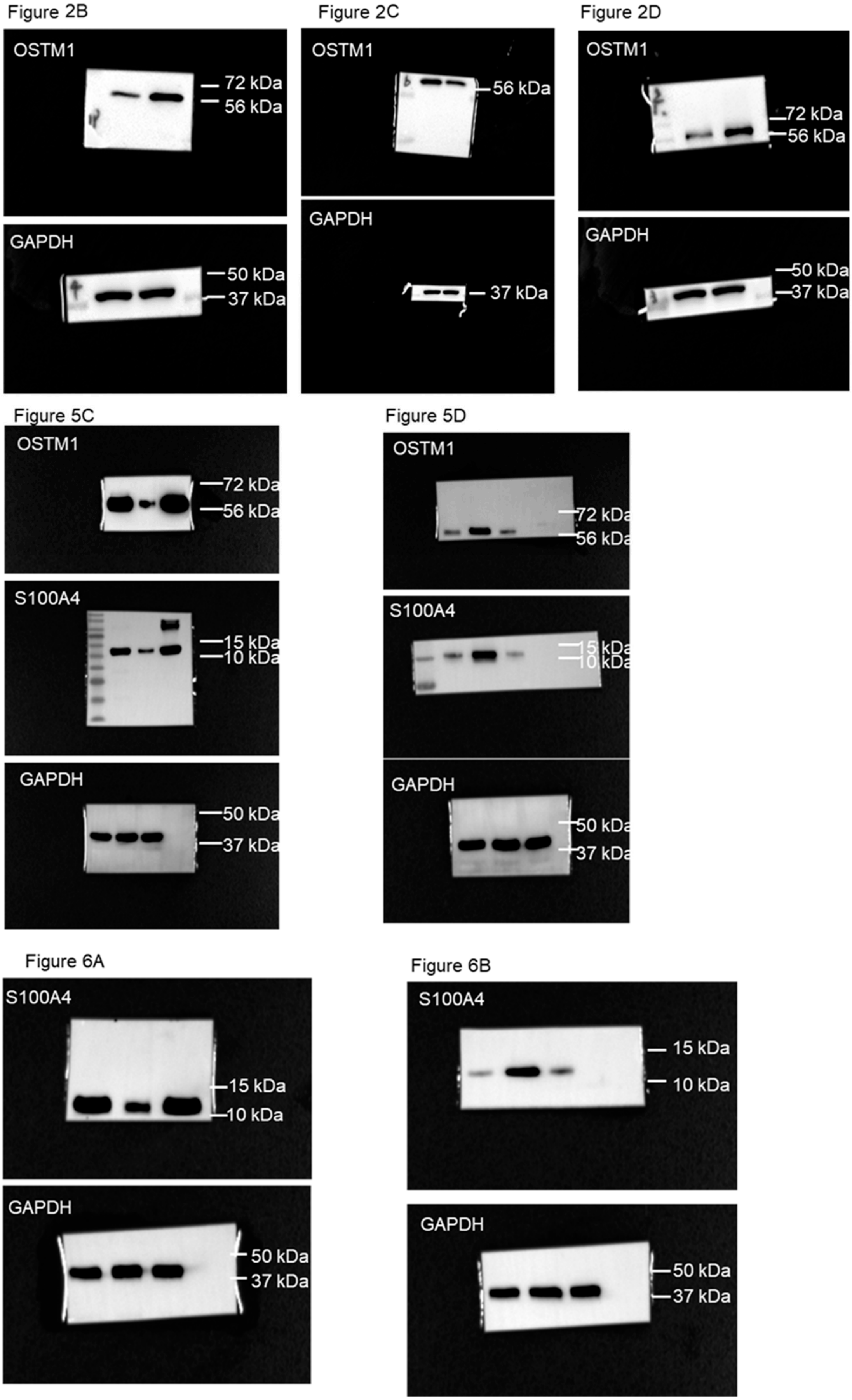

Figure S1. Uncropped gels for Western Blots in Figures.
